# Progress in Surgery

**Published:** 1902-03-29

**Authors:** 


### PROGRESS IN SURGERY.

(*Continued from page* 423.)

**Congenital Hydronephrosis.**—J. T. Shirlaw[6] records a case of this affection in an infant which he successfully operated upon when the child was nine weeks old. The infant was born with a swollen abdomen, which induced impeded delivery, and the swelling gradually increased until the date of operation. The swelling had the shape of a kidney and was dull on percussion and fluctuant, and occupied the left lumbar iliac, and hypochondriac regions, and passed to the right of the middle line. The bowels were crowded up under the liver. At first the descending colon could be felt and percussed over the surface of the tumour, but eventually it slipped round to the lumbar region. A four-inch incision was made rather further forward than in the ordinary position for nephrotomy, and the general peritoneal cavity opened. Then the thin wall of the cyst was tapped with a small trochar and cannula A pint and a half at least of fluid escaped. A drainage tube was fixed in with a suture passing through the skin, and another tube passed into the peritoneum. There was great collapse after the operation. A fistula formed, which finally closed at the end of five and a half months. The cause of the hydronephrosis was not sought for. Cure is attributed to the destruction of secreting tissue by (*a*) the enormous distension and (*b*) the post-operative suppuration in the sac.

**Cystic Kidney.**—J. Niemack (Iowa) has recorded a case of this affection.[7] He alludes to the differences between those cases in which one or two cysts are found, and which are now treated by excision of the cysts, and those cases in which the whole kidney substance is a conglomeration of large and small cysts. Some of the latter are found in new-born babes, and in all it seems that both kidneys are affected, though often in different degree. Niemack's patient was a woman of 43, and on an abdominal examination being made for another ailment a smooth kidney-shaped movable swelling, rather larger than a normal kidney, was discovered. Three months later she had several slight chills, and severe pain in the abdomen on the right side below the liver. The tumour was twice the size it had been at the previous examination, and had several elevations on its surface of nearly walnut size. The urine now contained a little albumen, mucus, pus, and blood, and had the specific gravity of 1015. Later, though the temperature continued irregular, the albumen disappeared. The diagnosis was made of a floating kidney with twisted pedicle, cystic degeneration, and septic infection. Lumbar nephrectomy was advised and performed, after a preliminary examination of both kidneys by laparotomy. Eighteen ounces of urine were passed in the first 24 hours after the operation, but after that vomiting set in and the urine became scanty. The patient died comatose 62 hours after the operation. No post-mortem was made. The removed organ was full of cysts. The writer alludes to the fact that the danger of nephrectomy decreases in proportion to the amount of increased work the remaining kidney has grown accustomed to perform, and the results may be better therefore in long-standing disease on one side than in more acute cases. The strangulation from twisting of the pedicle in this case had led to septic changes and infection of the cysts with the colon bacillus, but dilatation and inflammation of the pelvis was not present. Niemack concludes that nephrectomy for cystic disease of the kidney will nearly always end in death, and that in his case nephrorraphy would have prevented the complication which finally caused death. He suggests that the best treatment for the septic condition which had arisen would have been to remove the twist, split the kidney down to the pelvis, stitch the organ into position and drain with plain gauze. Thus septic material would then have found an outlet. The writer alludes to a case in which a woman of 50 had lived 12 years with highly developed bilateral cystic kidneys, and finally died accidentally after exploratory laparotomy. The case shows for how long a time such kidneys may do a goodly amount of work.

**Tumours of the Kidney.**—T. R. Jessop [8] relates his personal experiences in the operative treatment of renal tumours. He alludes to the danger in performing nephrectomy on the right side, of including a portion of the vena cava when ligaturing the pedicle, and mentions a case of his own in which this actually happened. The patient, a child aged nearly three years, had a movable renal tumour the size of a fœtal head, and weighing 3 lb. 2 oz., removed by the abdominal route. Five hours after the operation the pulse was good and the child was cheerful and chatting with the nurses; but two hours later still she died with absolute suddenness. Probably the death was from the formation of a thrombus where the vena cava had been ligatured, and pulmonary embolism. Jessop has performed nephrectomy 11 times in young children for tumours, nine times by the lumbar route, twice by the abdominal. One, a pallid, wasted child, 13 months old, died under the operation, another lived seven hours only, viz., the case quoted above. Nine recovered from the operation, but of these the longest survivor lived only two years and five months, whilst one died at the end of nine weeks. The writer remarks that better results can only be expected if diagnosis is made and operation undertaken earlier in the history of these cases. For the purpose an early intra-peritoneal inspection is suggested. Nephrectomy for tumour in adults has been performed by Jessop six times. Two recovered, two died from shock within a few hours, and the other two died in a few days.

**Nephrolithotomy.**—Jessop [9] states that in 1896 in a conversation with Mr. Henry Morris respecting a patient with advanced tubercular disease of the kidneys, that surgeon said he was in the habit of withdrawing the kidney from its bed, so as to fully expose it outside the lumbar wound, of splitting it into two symmetrical halves by an incision from its convex border through to the pelvis, with the object of curetting masses of tubercle, and of returning the kidney to its natural position after suturing it with three or four sutures passed deeply through its substance and tied just tightly enough to arrest bleeding. Since then Jessop has adopted the same procedure five times for the removal of stones from the kidney with most satisfactory results. In none was there any difficulty in controlling the hæmorrhage, all the wounds healed directly, and there was no urinary fistula to complicate the results. The procedure makes it much easier to examine the ureter from end to end by means of a probe.

[6] Lancet, Nov. 16, 1901, p. 1462. [7] Ann. of Surg., December, 1901, p. 811. [8] Lancet, Dec. 14, 1901, p. 1647. [9] Op. cit.

